# The Associations Between Fibrinogen and Septic Shock in Critically Ill Patients With Sepsis: A Retrospective Cohort Study

**DOI:** 10.1155/emmi/8849147

**Published:** 2026-03-01

**Authors:** Jianqin Huang, Murong Lu, Yu Zhai, Jiexuan Xu, Pengcheng Duan, Shuting Liu, Xuemei Liu, Hongjing Yu

**Affiliations:** ^1^ Intensive Care Unit, The Second Affiliated Hospital of Guangzhou Medical University, Guangzhou, Guangdong, China, gzhmc.edu.cn; ^2^ Department of Cardiovascular Medicine, The Second Affiliated Hospital of Guangzhou Medical University, Guangzhou, Guangdong, China, gzhmc.edu.cn; ^3^ Nursing Administration Department, The Second Affiliated Hospital of Guangzhou Medical University, Guangzhou, Guangdong, China, gzhmc.edu.cn

**Keywords:** fibrinogen, intensive care unit, MIMIC database, sepsis, septic shock

## Abstract

**Background:**

Fibrinogen has been used as a prognostic indicator for sepsis. However, the associations of fibrinogen and septic shock in septic patients remain unclear. This study aimed to explore the relationship between fibrinogen levels and the occurrence of septic shock in patients with sepsis.

**Methods:**

Data were retrospectively analyzed from the Medical Information Mart for Intensive Care IV (MIMIC‐IV v3.1) database. The Boruta algorithm and random forest model were used for feature selection to ensure the important variables affecting results. Multivariate logistic regression assessed the association between fibrinogen and septic shock. Subgroup analysis was conducted to evaluate the impact of additional variables on the results.

**Results:**

The study included 3302 septic patients. Fibrinogen was significantly associated with septic shock (odds ratio [OR] = 1.46; 95% confidence interval [CI], 1.35–1.56), and the risk of septic shock increased with higher fibrinogen levels (all *p* values for trend < 0.001). The ROC curve demonstrated the predictive accuracy of fibrinogen for septic shock in sepsis patients. After adjustment for demographics and laboratory, fibrinogen had a higher area under curve (AUC) value (0.78; 95% CI, 0.76–0.79) than SOFA (AUC, 0.58; 95% CI, 0.56–0.61), SASP II (AUC, 0.67; 95% CI, 0.65–0.69), and APS II (AUC, 0.69; 95% CI, 0.68–0.71).

**Conclusion:**

A linear relationship was found between fibrinogen levels and septic shock. Elevated fibrinogen levels were linked to a higher risk of septic shock in septic patients.

## 1. Introduction

Sepsis is a life‐threatening dysfunction of the organs caused by a dysregulated host response to infection [1]. A national epidemiological study conducted in 2020 reported an incidence of 20.6 cases of sepsis per 100 individuals in the intensive care unit (ICU), with a mortality rate of 35.5% [2]. Timely identification and diagnosis are crucial during the treatment, especially for patients with septic shock, who have a higher mortality rate [3]. However, sepsis patients exhibit a variety of symptoms and signs, posing challenges for medical staff to make an accurate prediction [4]. Furthermore, symptoms and signs in septic shock patients are subtle in the early disease stage, heightening diagnostic challenges.

Sepsis and septic shock were often accompanied by coagulation disorders, mainly due to the activation of intravascular coagulation and damage to the microvascular endothelium [5, 6]. Certain hemostatic biomarkers are considered risk factors for sepsis onset and death [7, 8]. Fibrinogen, a plasma glycoprotein and essential coagulation factor, is a key mediator of hemostasis and contributes to antimicrobial defense mechanisms [9, 10]. Fibrinogen can indicate the prognosis and severity of diseases. An observational cohort study demonstrated that fibrinogen levels can predict the severity of coronary artery disease in patients with type 2 diabetes. Elevated fibrinogen levels are associated with increased coronary artery anatomical complexity and a higher incidence of major adverse cardiovascular and cerebrovascular events [11]. In addition, elevated fibrinogen levels were associated with an increased all‐cause mortality rate in patients with coronary heart disease [12]. Nevertheless, no significant correlation was observed between elevated fibrinogen levels and the prognosis of septic patients [13]. In contrast, a study proposed that an increase in fibrinogen levels was associated with a decreased risk of 28 day all‐cause death in sepsis patients [14]. Elevated fibrinogen levels typically indicate the depletion of hemostatic factors and are associated with the hypercoagulable and hyperfibrinolytic states observed in sepsis‐induced coagulopathy [15]. There is an increase in fibrinogen synthesis in the plasma of patients with sepsis or septic shock [16]. Currently, the association between higher or lower fibrinogen levels and the occurrence of septic shock in sepsis remains insufficiently studied. Furthermore, the dose‐response relationship between them has not been fully elucidated.

This study aims to investigate the correlation between fibrinogen and the risk of septic shock in sepsis patients to provide a basis for the clinical assessment of patients’ conditions.

## 2. Methodology

### 2.1. Data Source

This observational cohort study utilized the Medical Information Mart for Intensive Care IV (MIMIC‐IV) database, version 3.1, a publicly accessible and extensive dataset designed for critical care research [17]. The MIMIC‐IV database comprises anonymized data from 2008 to 2024, sourced from the intensive care units of the Beth Israel Deaconess Medical Center, a leading academic medical center in Boston, USA [18, 19]. The MIMIC‐IV dataset encompasses detailed patient demographics, laboratory results, nursing notes, diagnostic records, medication logs, and additional critical health information. Given that all patient data have been de‐identified, the need for informed consent and additional ethical approval from individual patients is obviated. Our researchers have secured a license (record ID: 13578377) to use the database after completing the necessary training and assessments.

### 2.2. Study Population

**FIGURE 1:**
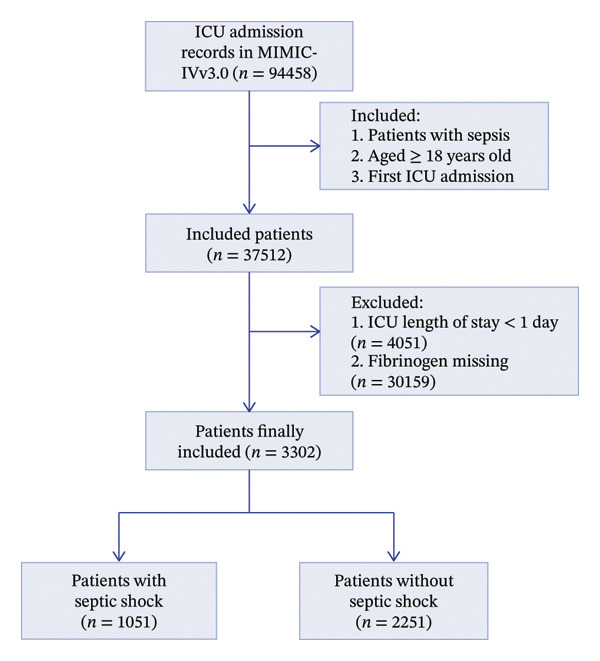
Flowchart of patients’ inclusion and exclusion. Abbreviation: ICU, intensive care unit.

This study enrolled patients aged ≥ 18 years who were admitted to the ICU for the first time and diagnosed with sepsis based on the Sepsis‐3 criteria. Sepsis‐3 is diagnosed as an increase of ≥ 2 points in the Sequential Organ Failure Assessment (SOFA) score when infection is suspected [1]. The exclusion criteria were (1) ICU stay < 1 day; (2) fibrinogen records were missing. Patients with sepsis were categorized into two groups based on whether they developed septic shock. The screening process is shown in Figure 1. This study followed the Strengthening the Reporting of Observational Studies in Epidemiology (STROBE) guidelines for reporting (Supporting Table 1).

### 2.3. Data Extraction

For this study, all data and information were sourced from the MIMIC‐IV database. This database was meticulously preprocessed by healthcare professionals using specialized techniques to document and store patient data, ensuring accuracy and confidentiality. The data collection process involved a structured and methodical approach, utilizing Structured Query Language (SQL) through Navicat Premium software, version 15.0.12 [20]. Patient demographics, including age, race, and ICU length of stay (ICU LOS), were extracted from MIMIC‐IV. The SQL programs provided by Johnson et al. were used to calculate the SOFA, simplified acute physiology score II (SAPS II), and acute physiology score III (APS III) [17]. Vital signs included heart rate (HR), respiratory rate (RR), systolic blood pressure (SBP), diastolic blood pressure (DBP), mean blood pressure (MBP), and blood oxygen saturation (SpO2). Laboratory indicators included fibrinogen, activated partial thromboplastin time (APTT), international normalized ratio (INR), prothrombin time (PT), creatinine, sodium, potassium, blood urea nitrogen (BUN), red blood cells (RBC), hemoglobin, hematocrit, red blood cell distribution width (RDW), white blood cells (WBC), platelets (PLT), lactate, total bilirubin, alanine aminotransferase (ALT), and aspartate aminotransferase (AST). Comorbidities included myocardial infarction, heart failure, chronic obstructive pulmonary disease (COPD), renal disease, liver disease, cerebrovascular disease, diabetes, and malignant tumors. Treatment measures included using vasoactive drugs, ventilation, continuous renal replacement therapy (CRRT), and enteral nutrition. Vital signs, laboratory results, disease severity scores, and treatment measures were extracted from the first measurements within 24 h after ICU admission (Table 1). Variables with missing data exceeding 30%, such as albumin and temperature, were excluded to reduce potential bias (Supporting Figure 1). For variables with a data missing rate of less than 30%, multiple imputations were performed using the “mice” package in R software with a random forest method.

**TABLE 1:** Baseline characteristics of patients with sepsis in the ICU.

Characteristics	Total	Nonseptic shock	Septic shock	*p* value
	(*N* = 3302)	(*N* = 2251)	(*N* = 1051)	
*General characteristics*				
Age, years	62.87 (51.43–73.60)	61.74 (50.50–72.32)	65.45 (53.73–75.98)	< 0.001
Race, *n* (%)				0.723
White	1946 (58.9)	1327 (59.0)	619 (58.9)	
Black	358 (10.8)	243 (10.8)	115 (10.9)	
Asian	129 (3.9)	93 (4.1)	36 (3.4)	
Hispanic	159 (4.8)	113 (5.0)	46 (4.4)	
Other	710 (21.5)	475 (21.1)	235 (22.4)	
Gender, *n* (%)				0.004
M	1924 (58.3)	1350 (60.0)	574 (54.6)	
F	1378 (41.7)	901 (40.0)	477 (45.4)	
ICU los, days	4.26 (2.35–8.97)	3.90 (2.25–7.62)	5.57 (2.78–12.27)	< 0.001

*Comorbidities or symptoms*				
Myocardial infarction, *n* (%)				0.518
NO	2817 (85.3)	1927 (85.6)	890 (84.7)	
YES	485 (14.7)	324 (14.4)	161 (15.3)	
Heart Failure, *n* (%)				< 0.001
NO	2510 (76.0)	1768 (78.5)	742 (70.6)	
YES	792 (24.0)	483 (21.5)	309 (29.4)	
Cerebrovascular Disease, *n* (%)				0.551
NO	2936 (88.9)	2007 (89.2)	929 (88.4)	
YES	366 (11.1)	244 (10.8)	122 (11.6)	
COPD, n (%)				0.007
NO	2583 (78.2)	1791 (79.6)	792 (75.4)	
YES	719 (21.8)	460 (20.4)	259 (24.6)	
Renal Disease, *n* (%)				0.047
NO	2587 (78.3)	1786 (79.3)	801 (76.2)	
YES	715 (21.7)	465 (20.7)	250 (23.8)	
Malignant cancer, *n* (%)				0.371
NO	2642 (80.0)	1791 (79.6)	851 (81.0)	
YES	660 (20.0)	460 (20.4)	200 (19.0)	
Liver disease, *n* (%)				0.445
NO	2025 (61.3)	1370 (60.9)	655 (62.3)	
YES	1277 (38.7)	881 (39.1)	396 (37.7)	
Diabetes, *n* (%)				0.01
NO	2410 (73.0)	1674 (74.4)	736 (70.0)	
YES	892 (27.0)	577 (25.6)	315 (30.0)	

*Vital signs*				
HR (bpm)	90.00 (77.25–104.00)	88.00 (76.00–102.00)	94.00 (80.00–108.00)	< 0.001
RR (times/min)	20.00 (16.00–24.00)	19.00 (16.00–23.00)	22.00 (18.00–26.00)	< 0.001
SPO2 (%)	98.00 (96.00–100.00)	98.00 (96.00–100.00)	97.00 (95.00–99.00)	< 0.001
SBP (mmHg)	110.00 (98.00–124.00)	113.00 (100.00–128.00)	104.00 (95.00–116.00)	< 0.001
DBP (mmHg)	60.00 (52.00–69.00)	61.00 (53.00–71.00)	57.00 (50.00–65.00)	< 0.001
MBP (mmHg)	74.00 (66.00–85.00)	76.00 (68.00–87.00)	71.00 (64.00–80.00)	< 0.001

*Laboratory parameters*				
Fibrinogen (mg/dL)	261.00 (173.00–426.00)	245.00 (169.50–377.00)	325.00 (181.50–513.00)	< 0.001
INR	1.50 (1.30–1.90)	1.40 (1.20–1.80)	1.70 (1.40–2.20)	< 0.001
PT, second	16.30 (13.80–20.80)	15.60 (13.60–19.20)	18.50 (14.90–24.40)	< 0.001
APTT, second	34.10 (29.20–44.00)	32.80 (28.50–40.65)	38.30 (31.00–50.45)	< 0.001
BUN (mg/dL)	24.00 (15.00–42.00)	22.00 (14.00–36.50)	32.00 (19.00–51.00)	< 0.001
Creatinine (mg/dL)	1.20 (0.80–2.10)	1.10 (0.80–1.80)	1.60 (1.00–2.70)	< 0.001
Sodium (mmol/L)	138.00 (135.00–141.00)	139.00 (136.00–142.00)	137.00 (134.00–141.00)	< 0.001
Potassium (mmol/L)	4.20 (3.80–4.70)	4.20 (3.80–4.70)	4.20 (3.70–4.80)	0.906
Hematocrit (%)	28.70 (24.50–33.80)	28.60 (24.20–33.50)	28.80 (24.90–34.10)	0.053
Hemoglobin (g/dL)	9.40 (8.00–11.10)	9.50 (8.00–11.10)	9.40 (8.00–11.10)	0.598
PLT, 10^9^/L	120.00 (71.00–195.00)	116.00 (71.00–187.00)	128.00 (71.00–208.00)	0.01
RBC (m/uL)	3.14 (2.64–3.74)	3.13 (2.64–3.73)	3.15 (2.63–3.74)	0.965
RDW (%)	15.60 (14.20–17.80)	15.40 (14.10–17.50)	16.00 (14.50–18.30)	< 0.001
WBC, 10^9^/L	11.70 (7.20–17.50)	11.00 (7.10–15.90)	13.80 (7.50–21.20)	< 0.001
Lactate (mmol/L)	2.70 (1.70–4.90)	2.40 (1.55–4.10)	3.30 (1.90–6.35)	< 0.001
ALT (IU/L)	45.00 (21.00–196.75)	45.00 (21.00–226.00)	48.00 (22.00–146.50)	0.351
AST (IU/L)	82.00 (37.00–388.75)	79.00 (36.00–416.50)	88.00 (39.00–322.00)	0.364
Total Bilirubin (g/dL)	1.50 (0.70–4.20)	1.40 (0.60–4.10)	1.60 (0.70–4.30)	0.031

*Medication or treatment*				
Vasoactive agents, *n* (%)				< 0.001
NO	1603 (48.5)	1330 (59.1)	273 (26.0)	
YES	1699 (51.5)	921 (40.9)	778 (74.0)	
Ventilation, *n* (%)				0.25
NO	733 (22.2)	513 (22.8)	220 (20.9)	
YES	2569 (77.8)	1738 (77.2)	831 (79.1)	
Enteral Nutrition, *n* (%)				< 0.001
NO	2930 (88.7)	2041 (90.7)	889 (84.6)	
YES	372 (11.3)	210 (9.3)	162 (15.4)	
CRRT, *n* (%)				< 0.001
NO	2971 (90.0)	2084 (92.6)	887 (84.4)	
YES	331 (10.0)	167 (7.4)	164 (15.6)	

*Disease severity scores*				
GCS	15.00 (14.00–15.00)	15.00 (14.00–15.00)	15.00 (14.00–15.00)	0.007
SAPS II	44.00 (34.00–56.00)	41.00 (32.00–51.00)	51.00 (40.00–63.00)	< 0.001
APS III	58.00 (43.00–77.00)	53.00 (39.00–70.00)	71.00 (54.00–89.00)	< 0.001
CCI	5.00 (3.00–7.00)	5.00 (3.00–7.00)	5.00 (3.00–8.00)	< 0.001
SOFA score	4.00 (3.00–6.00)	4.00 (2.00–6.00)	5.00 (3.00–7.00)	< 0.001

*Note:* Values are presented as median (interquartile range) or number (percent).

Abbreviations: APTT, activated partial thromboplastin time; bpm, beats per minute; BUN, blood urea nitrogen; COPD, chronic obstructive pulmonary disease; DBP, diastolic blood pressure; HR, heart rate; ICU LOS, icu length of stay; INR, international normalized ratio; MBP, mean blood pressure; PLT, platelet; PT, prothrombin time; RBC, red blood cell; RDW, red cell distribution width; RR, respiratory rate; SBP, systolic blood pressure; SpO2, blood oxygen saturation; WBC, white blood cell.

### 2.4. Primary Outcome

This study primarily aimed to evaluate the occurrence of septic shock during ICU stay. Septic shock was diagnosed as persistent hypotension requiring vasopressors to maintain a mean arterial pressure ≥ 65 mmHg after adequate fluid resuscitation, along with elevated blood lactate levels > 2 mmol/L [21].

### 2.5. Statistical Analysis

This study used the R program for statistical analysis (version 4.4.1). Baseline characteristics were presented as means ± standard deviations (SD) for normally distributed quantitative data, medians (interquartile ranges, IQR) for skewed data, and numbers (percentages) for categorical data. The Shapiro–Wilk test was used to assess the normal distribution of continuous variables. For normally distributed quantitative data, comparisons were made using the independent *t*‐test, and for skewed data, the Mann–Whitney test was used. Categorical data were compared using the chi‐square test or Fisher’s exact test. Moreover, exploration of linear correlation between variables using Spearman correlation for skewed data and Pearson correlation for the normal distribution of continuous variables.

To investigate the association between fibrinogen and septic shock in sepsis patients, we employed machine learning methods to select features based on prior literature and assess their importance in predictive models. The Boruta algorithm, a widely used feature selection method, is chosen for its significance in this situation. The algorithm operates on two key principles: “shadow features” and the “binomial distribution”. Specifically, the Boruta algorithm generates a set of replicated segments from the original data, referred to as shadow features. A feature is considered statistically significant and retained if its Z‐score exceeds the highest Z‐score of the shadow features. Conversely, features with Z‐scores below this threshold are excluded from further analysis [22]. For feature selection, we used the random forest algorithm, and Shapley Additive Explanations (SHAP) values were applied to visualize the importance of each feature. SHAP values improve the interpretability of machine learning models by mitigating their “black box” nature, enabling clinical practitioners to understand the model’s outcomes better [23].

Univariate and multivariate logistic regression analyses were used to examine the relationship between fibrinogen and the incidence of septic shock. The odds ratio (OR) and its corresponding 95% confidence interval (CI) were calculated. Three models were established: Model 1 included fibrinogen without adjustment for covariates; Model 2 added patient characteristic variables including age, heart failure, and liver disease; and Model 3, which extends Model 2, incorporates variables selected according to their significance using the Boruta algorithm, including HR, RR, SpO2, SBP, DBP, MBP, PT, APTT, INR, BUN, creatinine, sodium, potassium, hemoglobin, hematocrit, PLT, RBC, RDW, WBC, lactate, total bilirubin, ALT, and AST (Figure 2).

FIGURE 2Application of machine learning in feature selection. (a) Assessment of variable importance using the Boruta algorithm. (b) Assessment of variable importance using Shapley Additive Explanations (SHAP) computed by the random forest model.

**Figure:**
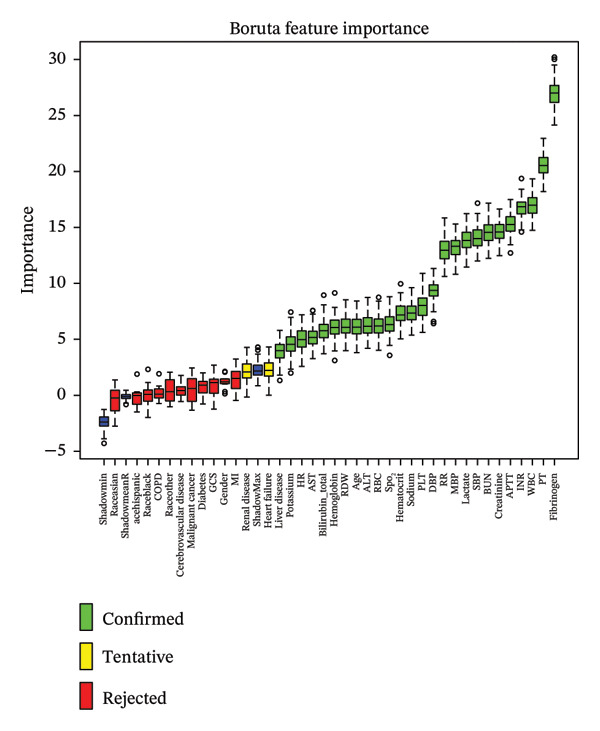
(a)

**Figure:**
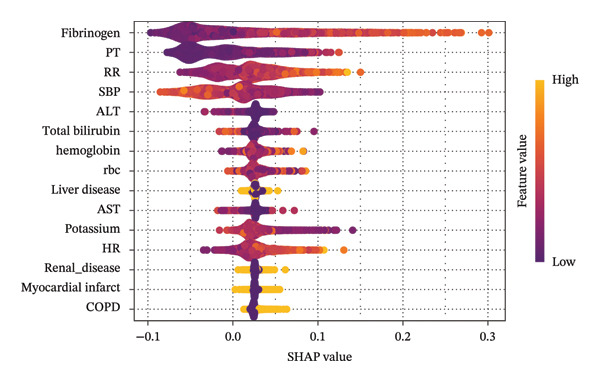
(b)

Four‐knot restricted cubic spline (RCS) curves were utilized to examine the nonlinear relationship between fibrinogen and the incidence of septic shock. Subsequently, the predictive accuracy of fibrinogen for septic shock was assessed using receiver operating characteristic (ROC) curves and the area under the ROC curve (AUC). Bootstrap for internal validation and calibration curve plots were constructed to quantify calibration accuracy. Additionally, subgroup analyses were conducted to evaluate interactions and verify fibrinogen’s influence on septic shock incidence within each subgroup.

### 2.6. Sensitivity Analysis

To evaluate the robustness of our findings, sensitivity analyses were conducted. Given that comorbidities such as chronic pulmonary disease and renal disease are associated with heightened susceptibility to septic shock, the analysis focused specifically on patients without these conditions.

## 3. Results

### 3.1. Sociodemographic and Clinical Characteristics of Patients

This retrospective study included 3302 patients diagnosed with sepsis, and their baseline characteristics are presented in Table 1. Among the participants, 1051 individuals experienced sepsis‐induced shock, while 2251 individuals did not experience shock. In addition, 1924 were male (58.3%) and 1378 were female (41.7%), and the median age was 62.87 years. The majority of the patients (58.9%) were White. Compared with the nonshock group, patients in the shock group were older and had significantly lower blood pressure (*p* < 0.001). The incidences of heart failure, COPD, renal disease, and diabetes mellitus were significantly higher in the shock group than in the nonshock group (*p* < 0.01). Furthermore, higher levels of SOFA, SAPS II, and APS III were observed in the shock group compared with the nonshock group (*p* < 0.001). In addition, only total bilirubin and fibrinogen showed a negative correlation (Supporting Figure 2).

### 3.2. Association Between Fibrinogen and Septic Shock

**FIGURE 3:**
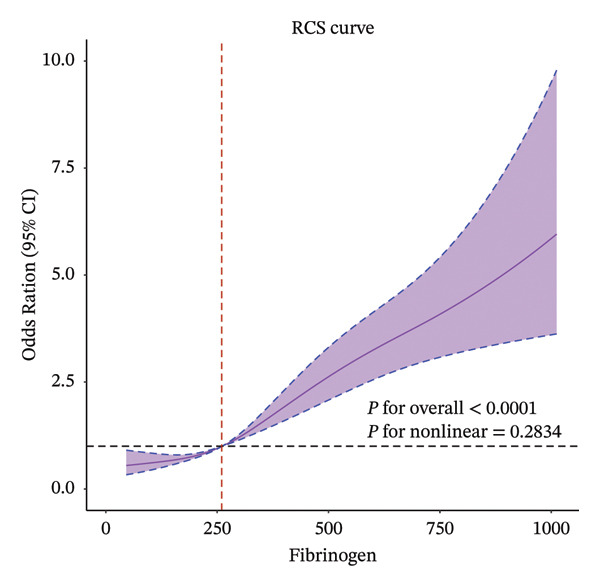
Evaluation of the nonlinear relationship between fibrinogen and outcomes using restricted cubic spline (RCS) curves.

**FIGURE 4:**
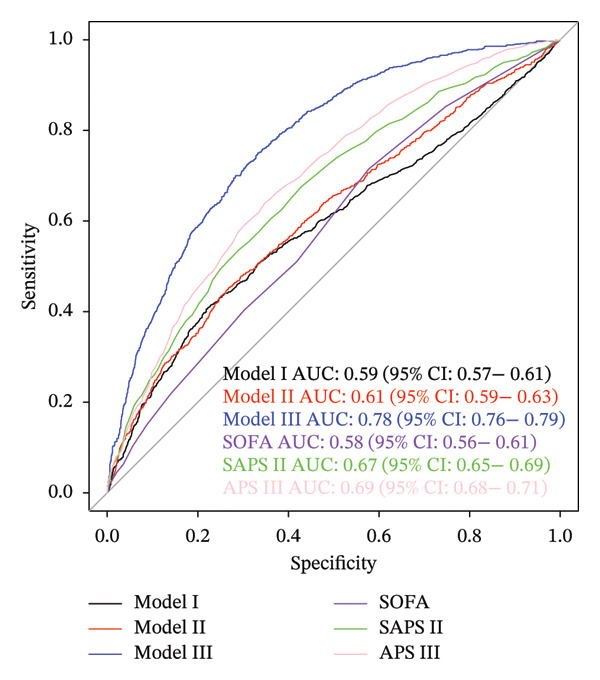
Receiver operating characteristic (ROC) curve analysis evaluated the value of the fibrinogen in assessing the risk of septic shock in sepsis patients and compared the differences among models. Data are presented as AUC (95% CI). Model 1 included fibrinogen without adjustment for covariates. Model 2 added patient characteristic variables including age, heart failure, and liver disease. Model 3, which extends Model 2, incorporates variables selected based on their importance using the Boruta algorithm, including HR, RR, SpO2, SBP, DBP, MBP, PT, APTT, INR, BUN, creatinine, sodium, potassium, hemoglobin, hematocrit, PLT, RBC, RDW, WBC, lactate, total bilirubin, ALT, and AST. Abbreviations: AUC, area under the curve; CI, confidence interval.

**FIGURE 5:**
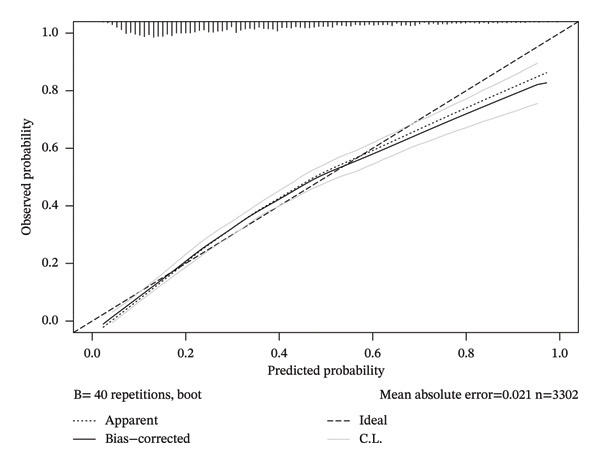
Calibration curve analysis of Model 3.

The findings from all three developed models consistently indicated that fibrinogen is an independent risk factor for septic shock in sepsis patients. In the multivariate logistics regression model (Model 3), the model’s AUC was 0.78 (95% CI, 0.76–0.79). For fibrinogen (continuous variable), the OR value was 1.69 (95% CI, 1.54–1.89), with an optimal cutoff value of 338.55 mg/dL, a sensitivity of 74.97%, and a specificity of 66.77%. In Model 1, the AUC was 0.59 (95% CI, 0.57–0.61), the OR value was 1.455 (95% CI, 1.35–1.56), the optimal cutoff value was 388.55 mg/dL, the sensitivity was 40.53%, and the specificity was 78.14%. The internal validation results showed that Model 3 had an AUC of 0.761, a sensitivity of 88.46%, and a specificity of 39.21%. When fibrinogen levels were divided into three groups for comparison in the logistics analysis, patients with fibrinogen levels exceeding 400 mg/dL exhibited a significantly elevated risk of septic shock compared to those below this threshold. All three models demonstrated that an upward trend in sepsis risk was consistently observed with increasing fibrinogen levels (*P* for trend < 0.001) (Table 2). The RCS analysis revealed a linear association between septic shock and fibrinogen (Figure 3). When fibrinogen levels were around 250–270 mg/dL, the OR of fibrinogen was close to 1, suggesting that elevated fibrinogen levels are associated with an increased risk of septic shock. Compared to the SOFA score, fibrinogen in Model 1 showed superior predictive performance (Figure 4). Model 3 showed better predictive ability than SAPS II and APS II when combined with other variables. The calibration curve shows that Model 3 has great calibration (Figure 5).

**TABLE 2:** The association between different fibrinogen levels and occurrence of septic shock.

	Model1	Model2	Model3
Fibrinogen (mg/dL)	1.46 (1.35–1.56)	1.43 (1.33–1.54)	1.69 (1.54–1.89)
Fibrinogen (mg/dL)			
< 150	Ref	Ref	Ref
150–400	0.75 (0.61–0.92)	0.71 (0.57–0.89)	1.21 (0.94–1.57)
> 400	1.95 (1.56–2.44)	1.82 (1.45–2.29)	3.26 (2.42–4.41)
*P* for trend	< 0.001	< 0.001	< 0.001

*Note:* Data are presented as OR (95% CI). Model 1: included fibrinogen without adjustment for covariates. Model 2: adjusted for age, heart failure, and liver disease. Model 3: adjusted for age, heart failure, and liver disease, HR, RR, SpO2, SBP, DBP, MBP, PT, APTT, INR, BUN, creatinine, sodium, potassium, hemoglobin, hematocrit, PLT, RBC, RDW, WBC, lactate, total bilirubin, ALT, and AST.

Abbreviations: CI = confidence interval, OR = odds ratio, Ref = reference.

### 3.3. Subgroup Analysis and Sensitivity Analysis

A subgroup analysis was conducted to evaluate the impact of baseline characteristics on fibrinogen levels in sepsis patients experiencing septic shock in Model 3 (Table 3). The sensitivity analysis shows the result was consistent across different models and adjustments (Supporting Table 2).

**TABLE 3:** Subgroup analysis of the multivariable‐adjusted odds ratio (OR) pertaining to the association between fibrinogen and the occurrence of septic shock on Model 3.

Variable	Count	Percent (%)	Fibrinogen ≤ 150	Fibrinogen = 150–400	*p* value	Fibrinogen ≥ 400	*p* value	*P* for interaction
Age, yr								0.241
18–60	1403	42.5	Ref	0.74 (0.55–1.00)	0.047	1.57 (1.12–2.19)	0.008	
≥ 60	1899	57.5	Ref	0.71 (0.52–0.96)	0.026	2.02 (1.47–2.77)	< 0.001	
Gender								0.130
Male	1924	58.3	Ref	0.79 (0.59–1.05)	0.103	2.33 (1.72–3.15)	< 0.001	
Female	1378	41.7	Ref	0.70 (0.51–0.96)	0.025	1.55 (1.10–2.16)	0.011	
Myocardial infarction								0.811
NO	2817	85.3	Ref	0.73 (0.58–0.91)	0.006	1.92 (1.51–2.45)	< 0.001	
YES	485	14.7	Ref	0.88 (0.49–1.59)	0.679	2.14 (1.16–3.97)	< 0.001	
Heart Failure								0.310
NO	2510	76.0	Ref	0.68 (0.53–0.86)	0.001	1.80 (1.39–2.32)	< 0.001	
YES	792	24.0	Ref	1.00 (0.63–1.60)	0.994	2.28 (1.41–3.69)	0.001	
Renal disease								0.261
No	2587	78.3	Ref	0.71 (0.57–0.90)	0.004	1.76 (1.37–2.26)	< 0.001	
YES	715	21.7	Ref	0.92 (0.54–1.57)	0.771	2.82 (1.63–4.88)	< 0.001	
Liver disease								0.833
No	2025	61.3	Ref	0.86 (0.59–1.25)	0.425	2.38 (1.64–3.47)	< 0.001	
YES	1277	38.7	Ref	0.76 (0.58–1.00)	0.046	2.03 (1.35–3.04)	0.001	
CCI								0.453
1–3	962	29.1	Ref	0.63 (0.42–0.94)	0.025	1.57 (1.04–2.38)	0.033	
≥ 4	2340	70.9	Ref	0.79 (0.62–1.01)	0.062	2.16 (1.66–2.82)	< 0.001	

*Note:* Results are presented as OR (95% CI).

Abbreviations: CI, confidence interval; CCI, Charlson Comorbidity Index; Ref, reference.

## 4. Discussion

This study investigated the impact of fibrinogen on the risk of developing septic shock in patients with sepsis. Using data from the MIMIC database and a retrospective study, we demonstrated the association between fibrinogen with septic shock. The results indicate a significant correlation between fibrinogen levels and the incidence of septic shock, showing that as fibrinogen levels increase, the risk of septic shock also rises substantially. This finding underscores the critical role of coagulation responses in sepsis and its progression.

Fibrinogen is a readily accessible laboratory marker in clinical practice, typically elevated in response to systemic inflammation, tissue injury, and various types of cancer. This elevation is often implicated in thrombosis and vascular injury. Septic shock represents a severe progression of sepsis. In this section, pathogen‐associated molecular patterns activate Toll‐like receptors, trigger the NF‐κB pathway and release proinflammatory cytokines such as IL‐6 and TNF‐α, which in turn cause endothelial damage and excessive immune cell activation [24]. Fibrinogen can bind to integrin receptors on the surface of leukocytes, such as αMβ2 and αXβ2, thereby triggering cellular activation and consequently enhancing their phagocytic function [25]. Nevertheless, it also facilitates the migration of leukocytes and their movement across endothelial cells, activating the NF‐κB transcription factor [26]. This activation subsequently leads to the increased production of inflammatory cytokines and worsens endothelial damage [27]. In response to inflammatory mediators like IL‐6 and TNF‐α, the liver synthesizes increased amounts of fibrinogen, leading to elevated plasma concentrations. High fibrinogen levels contribute to infection containment by forming fibrin barriers that limit pathogen dissemination and enhance macrophage phagocytic activity [26]. While this process helps prevent the spread of infection, excessive coagulation activation can lead to microthrombosis, impairing organ microcirculation and worsening disease severity. In sepsis, persistent coagulation activation may result in disseminated intravascular coagulation, causing significant fibrinogen depletion and a subsequent hypocoagulable state [16]. As fibrinogen levels decline, the body’s capacity to maintain hemostasis becomes compromised, thereby elevating the risk of hemorrhage and potentially precipitating irreversible organ failure. Consequently, this exacerbates the severity of sepsis or leads to a poorer prognosis in septic shock [28]. This highlights that fibrinogen is strongly associated with uncontrolled inflammation, coagulation disorders, and organ dysfunction and serves as a reliable indicator for indicating the severity, aligning with the findings of this study.

In this retrospective cohort study, we observed a linear relationship between fibrinogen levels and septic shock incidence in sepsis patients. Yao et al. have suggested that elevated fibrinogen levels reflect the acute‐phase defense response of the body against infection, with high fibrinogen concentrations being associated with a lower 28 day mortality rate [14]. This indicates that patients who can mount an effective immune response upon infection may have better clinical outcomes. Although fibrinogen elevation may be a reactive response to sepsis, it also holds potential value in disease progression. A prospective study demonstrated that fibrinogen levels effectively distinguish sepsis from septic shock, which is consistent with our findings [29]. In our study, the identified cut‐off range was 3.38–3.88 g/dL, similar to the previously reported fibrinogen classification but different in sensitivity. This difference may be attributed to the higher prevalence of liver disease among septic shock patients and the prior use of tigecycline in their treatment [30]. Given that impaired liver function reduces fibrinogen synthesis, the observed cutoff value in our study was relatively lower. Nevertheless, our findings indicate that even when fibrinogen levels fall within the range of 3.38–3.88 g/L, below the previously reported threshold of 4.7 g/L, septic patients still exhibit a considerable risk of progressing to septic shock. This underscores the need for heightened clinical vigilance among healthcare professionals when managing patients with fibrinogen levels within this range.

The protective effect of normal‐range fibrinogen appears threshold‐dependent. As an acute‐phase reactant, moderate increases in fibrinogen during early infection likely reflect effective inflammation control and exert protective effects [31]. Low fibrinogen levels, indicating severe coagulation impairment, are linked to DIC‐related organ failure and increased mortality risk [14, 16]. This discrepancy may be due to the timing of fibrinogen measurement relative to disease progression [29]. Tuan et al. revealed that impaired fibrinogen synthesis in children results in a hypocoagulable state in patients, which exacerbates the severity of septic shock [32]. This suggests the protective effect may mask true risk differentiation. The actual protection may be concentrated at 150–338.55 mg/dL, while levels near 338.55 mg/dL may indicate a transition to a pathological state. After adjusting for coagulation indices, inflammatory markers, and other parameters in Model 3, the “protective effect” disappeared, indicating confounding by microthrombosis and inflammation severity [7, 33, 34]. Future studies should validate the 338.55 mg/dL threshold’s biological significance and explore its molecular mechanisms.

Feature selection is a crucial step in constructing predictive models. In this study, the Boruta algorithm was utilized for variable screening. This algorithm, based on random forests, systematically evaluates the importance of each variable by comparing “shadow features” with actual features [22]. Consequently, it significantly enhances the model’s accuracy, stability, and interpretability while reducing the risk of overfitting. This method assessed feature importance via the random forest model, and SHAP values were employed for visualization. Derived from cooperative game principles, SHAP values allocate precise contribution values to each feature, offering a more nuanced analysis of feature impact. This enables even complex models to be understood by clinical practitioners. Model 3 in this study identified multiple indicators, such as PT, APTT, INR, BUN, creatinine, sodium, potassium, hemoglobin, hematocrit, PLT, RBC, RDW, WBC, lactate, total bilirubin, ALT, and AST, as key factors in the development of septic shock in sepsis patients. Meanwhile, among individuals aged over 60, there is a correlation between high fibrinogen levels and the occurrence of septic shock, but no interaction effect is observed. This finding deepens our understanding of the pathophysiological mechanisms underlying sepsis progression and strongly supports using these indicators for risk stratification, early intervention, and management in clinical settings.

Our study has several limitations. First, as a retrospective study, it is inherently subject to certain biases. It is essential to note that the design of this study is limited to identifying associations, not establishing causal relationships. However, we attempted to minimize potential bias by adjusting for confounding factors as much as possible during data analysis. Second, fibrin degradation products play a crucial role in the progression of sepsis. Due to the limitations of retrospective clinical study, comprehensive data on fibrin degradation products were not available from the database, making it difficult to evaluate their impact on disease severity in septic patients. In addition, early fluid resuscitation may influence fibrinogen levels and affect the severity. However, determining the optimal dosage and timing of early fluid resuscitation to restore the therapeutic process remains challenging and requires further research. Various pathogens can trigger sepsis, each with distinct pathogenic mechanisms, eliciting different immune responses and exerting varying effects on the coagulation system. These differences merit further exploration. Meanwhile, fibrinogen is influenced by liver function and inflammatory mediators in patients, and the occurrence of septic shock is also related to the source of infection, pathogen, antibiotics used, DIC status, thromboelastography, CRP, and procalcitonin. Therefore, prospective multicenter clinical trials are needed to further validate our findings.

## 5. Conclusion

In patients with sepsis, fibrinogen levels exhibit a linear relationship with the risk of developing septic shock. Elevated fibrinogen levels are associated with an increased likelihood of septic shock. However, there are limitations in the present study, and large‐sample multicenter clinical trials are needed to further validate our findings in the future, aiding in risk stratification and early management.

## Author Contributions

All authors contributed to the study conception and design. Jianqin Huang, Murong Lu, and Hongjing Yu designed the study. Jianqin Huang drafted the manuscript. Yu Zhai curated the data from the MIMIC‐IV database. Jiexuan Xu and Xuemei Liu performed the formal analysis. Pengcheng Duan supported the methodology. Murong Lu was responsible for supervision. Shuting Liu was responsible for visualization.

## Funding

The authors received no specific funding for this work.

## Disclosure

All authors reviewed and edited the manuscript. All authors read and approved the final manuscript.

## Ethics Statement

The MIMIC‐IV database is an anonymized, publicly accessible resource. The project has been approved by the institutional review boards of the Massachusetts Institute of Technology (MIT) and Beth Israel Deaconess Medical Center (BIDMC), with informed consent requirements waived.

## Conflicts of Interest

The authors declare no conflicts of interest.

## Supporting Information

Supporting Table 1. The STROBE reporting checklist.

Supporting Table 2. The Sensitivity analysis between fibrinogen and occurrence of septic shock.

Supporting Figure 1. The miss rate of feature extraction in MIMIC‐IV databases.

Supporting Figure 2. Spearman correlation among fibrinogen and BUN, creatine, ALT, AST, total bilirubin.

## Supporting information


**Supporting Information** Additional supporting information can be found online in the Supporting Information section.

## Data Availability

All datasets used during the present study are publicly available in the MIMIC‐IV v3.1 database (https://physionet.org/content/mimiciv/3.1). The codes used in the manuscript are available from https://github.com/MITLCP/mimic-IV.
