# Supplementary material for: The Associations Between Fibrinogen and Septic Shock in Critically Ill Patients With Sepsis: A Retrospective Cohort Study
Source: Emerg Med Int. 2026 Mar 1;2026:8849147. doi: 10.1155/emmi/8849147 (PMC12951003; doi:10.1155/emmi/8849147)
Supplement: Supplementary file 1 — Supporting Information Additional supporting information can be found online in the Supporting Information section. [file EMMI-2026-8849147-s001.docx]

**Supplementary Table 1.** The STROBE reporting checklist

|  | Item Description | Location (or reason for not reporting) |
| --- | --- | --- |
| **Title and abstract** |  |  |
| [1a. Indicate the study’s design](https:/resources.equator-network.org/reporting-guidelines/strobe/items/title-abstract-indicate-study-design.html) | Indicate the study’s design with a commonly used term in the title or the abstract. | Absract, method |
| [1b. Abstract](https:/resources.equator-network.org/reporting-guidelines/strobe/items/abstract.html) | Provide in the abstract an informative and balanced summary of what was done and what was found. | Abstract |
| **Introduction** |  |  |
| [2. Background / rationale](https:/resources.equator-network.org/reporting-guidelines/strobe/items/background-rationale.html) | Explain the scientific background and rationale for the investigation being reported. | Background; paragraph 1 & 2 |
| [3. Objectives](https:/resources.equator-network.org/reporting-guidelines/strobe/items/objectives.html) | State specific objectives, including any prespecified hypotheses. | Background; paragraph 3 |
| **Methods** |  |  |
| [4. Study design](https:/resources.equator-network.org/reporting-guidelines/strobe/items/study-design.html) | Present key elements of study design early in the paper. | Methodology, Data source & study population;  paragraph 1 |
| [5. Setting](https:/resources.equator-network.org/reporting-guidelines/strobe/items/setting.html) | Describe the setting, locations, and relevant dates, including periods of recruitment, exposure, follow-up, and data collection. | Methodology, Data source & study population;  paragraph 1 |
| [6a. Eligibility criteria](https:/resources.equator-network.org/reporting-guidelines/strobe/items/eligibility-criteria.html) | **Cohort study:** Give the eligibility criteria, and the sources and methods of selection of participants. Describe methods of follow-up. **Case-control study:** Give the eligibility criteria, and the sources and methods of case ascertainment and control selection. Give the rationale for the choice of cases and controls. **Cross-sectional study:** Give the eligibility criteria, and the sources and methods of selection of participants. | Methodology, Data source ;  paragraph 1 |
| [6b. Matching criteria](https:/resources.equator-network.org/reporting-guidelines/strobe/items/matching-criteria.html) | **Cohort study:** For matched studies, give matching criteria and number of exposed and unexposed. **Case-control study:** For matched studies, give matching criteria and the number of controls per case. | Not applicable |
| [7. Variables](https:/resources.equator-network.org/reporting-guidelines/strobe/items/variables.html) | Clearly define all outcomes, exposures, predictors, potential confounders, and effect modifiers. Give diagnostic criteria, if applicable. | Methodology, Primary outcomes & Data extraction |
| [8. Data sources / measurement](https:/resources.equator-network.org/reporting-guidelines/strobe/items/data-sources-measurement.html) | For each variable of interest give sources of data and details of methods of assessment (measurement). Describe comparability of assessment methods if there is more than one group. | Methodology, Statistical analysis; paragraph 1-4 |
| [9. Bias](https:/resources.equator-network.org/reporting-guidelines/strobe/items/bias.html) | Describe any efforts to address potential sources of bias. | Methodology, Sensitivity analysis & Figure 2; paragraph 1 |
| [10. Study size](https:/resources.equator-network.org/reporting-guidelines/strobe/items/study-size.html) | Explain how the study size was arrived at. | Methodology, Statistical analysis; paragraph 1 & 2 |
| [11. Quantitative variables](https:/resources.equator-network.org/reporting-guidelines/strobe/items/quantitative-variables.html) | Explain how quantitative variables were handled in the analyses. If applicable, describe which groupings were chosen, and why. | Methodology, Statistical analysis; paragraph 1 & 2 |
| [12a. Statistical methods](https:/resources.equator-network.org/reporting-guidelines/strobe/items/statistical-methods-description.html) | Describe all statistical methods, including those used to control for confounding. | Methodology, Statistical analysis; paragraph 1-4 |
| [12b. Statistical methods – subgroups and interactions](https:/resources.equator-network.org/reporting-guidelines/strobe/items/statistical-methods-subgroups-interactions.html) | Describe any methods used to examine subgroups and interactions. | Methodology, Statistical analysis; paragraph 4 |
| [12c. Statistical methods – missing data](https:/resources.equator-network.org/reporting-guidelines/strobe/items/statistical-methods-missing-data.html) | Explain how missing data were addressed. | Methodology, Data extraction;  paragraph 1 |
| [12di. Statistical methods – loss to follow-up](https:/resources.equator-network.org/reporting-guidelines/strobe/items/statistical-methods-loss-to-follow-up.html) | **Cohort study:** If applicable, describe how loss to follow-up was addressed. | Not applicable |
| [12dii. Statistical methods – matching cases and controls](https:/resources.equator-network.org/reporting-guidelines/strobe/items/statistical-methods-matching-cases-controls.html) | **Case-control study:** If applicable, explain how matching of cases and controls was addressed. | Not applicable |
| [12diii. Statistical methods – sampling strategy](https:/resources.equator-network.org/reporting-guidelines/strobe/items/statistical-methods-analytical-methods-sampling-strategy.html) | **Cross-sectional study:** If applicable, describe analytical methods taking account of sampling strategy. | Not applicable |
| [12e. Statistical methods – sensitivity analyses](https:/resources.equator-network.org/reporting-guidelines/strobe/items/statistical-methods-sensitivity-analyses.html) | Describe any sensitivity analyses. | Methodology, Sensitivity analysis; paragraph 1 |
| **Results** |  |  |
| [13a. Participant numbers](https:/resources.equator-network.org/reporting-guidelines/strobe/items/participants-numbers.html) | Report the numbers of individuals at each stage of the study—e.g., numbers potentially eligible, examined for eligibility, confirmed eligible, included in the study, completing follow-up, and analysed; Consider use of a flow diagram. | Results, Sociodemographic and clinical characteristics of patients |
| [13b. Participants – non-participation](https:/resources.equator-network.org/reporting-guidelines/strobe/items/participants-non-participation.html) | Give reasons for non-participation at each stage. | Figure 1 |
| [13c. Participants – flow diagram](https:/resources.equator-network.org/reporting-guidelines/strobe/items/participants-flow-diagram.html) | Consider use of a flow diagram. | Figure 1 |
| [14a. Descriptive data – participant characteristics](https:/resources.equator-network.org/reporting-guidelines/strobe/items/descriptive-data-participant-characteristics.html) | Give characteristics of study participants (e.g., demographic, clinical, social) and information on exposures and potential confounders. Present the information in a table. | Results, Sociodemographic and clinical characteristics of patients |
| [14b. Descriptive data – missing data](https:/resources.equator-network.org/reporting-guidelines/strobe/items/descriptive-data-missing-data.html) | Indicate the number of participants with missing data for each variable of interest. | Methodology, Data extraction;  paragraph 1 |
| [14c. Descriptive data – follow-up time](https:/resources.equator-network.org/reporting-guidelines/strobe/items/descriptive-data-follow-up-time.html) | **Cohort study:** Summarise follow-up time—e.g., average and total amount. | Methodology, Primary outcomes |
| [15. Outcome data](https:/resources.equator-network.org/reporting-guidelines/strobe/items/outcome-data.html) | **Cohort study:** Report numbers of outcome events or summary measures over time. **Case-control study:** Report numbers in each exposure category, or summary measures of exposure. **Cross-sectional study:** Report numbers of outcome events or summary measures. | Table 1 |
| [16a. Main results](https:/resources.equator-network.org/reporting-guidelines/strobe/items/main-results.html) | Give unadjusted estimates and, if applicable, confounder-adjusted estimates and their precision (e.g., 95% confidence intervals). Make clear which confounders were adjusted for and why they were included. | Table 2, Fiure 3 &4 |
| [16b. Main results – category boundaries](https:/resources.equator-network.org/reporting-guidelines/strobe/items/main-results-category-boundaries.html) | Report category boundaries when continuous variables were categorised. | Table 2 |
| [16c. Main results – risk](https:/resources.equator-network.org/reporting-guidelines/strobe/items/main-results-risk.html) | If relevant, consider translating estimates of relative risk into absolute risk for a meaningful time period. | Table 2 |
| [17. Other analyses](https:/resources.equator-network.org/reporting-guidelines/strobe/items/other-analyses.html) | Report other analyses done—e.g., analyses of subgroups and interactions, and sensitivity analyses. | Table 3 and Figure 4; Supplementary Fig 1 and Table 2 |
| **Discussion** |  |  |
| [18. Key results](https:/resources.equator-network.org/reporting-guidelines/strobe/items/key-results.html) | Summarise key results with reference to study objectives. | Discussion;  paragraph 1 |
| [19. Limitations](https:/resources.equator-network.org/reporting-guidelines/strobe/items/limitations.html) | Discuss limitations of the study, taking into account sources of potential bias or imprecision. Discuss both direction and magnitude of any potential bias. | Discussion;  paragraph 6 |
| [20. Interpretation](https:/resources.equator-network.org/reporting-guidelines/strobe/items/interpretation.html) | Give a cautious overall interpretation considering objectives, limitations, multiplicity of analyses, results from similar studies, and other relevant evidence. | Discussion;  paragraph 2-5 |
| [21. Generalisability](https:/resources.equator-network.org/reporting-guidelines/strobe/items/generalisability.html) | Discuss the generalisability (external validity) of the study results. | Discussion;  paragraph 5 |
| **Other information** |  |  |
| [22. Funding](https:/resources.equator-network.org/reporting-guidelines/strobe/items/funding.html) | Give the source of funding and the role of the funders for the present study and, if applicable, for the original study on which the present article is based. | Not applicable |

| **Supplementary Table 2.** The Sensitivity analysis between fibrinogen and occurrence of septic shock | | | | |
| --- | --- | --- | --- | --- |
|  | Fibrinogen  (continuous) | Fibrinogen ≤ 150 | Fibrinogen=150-400 | Fibrinogen ≥ 400 |
| Unadjusted | 1.46 (1.35-1.56) | Ref | 0.75 (0.61-0.92) | 1.95 (1.56-2.44) |
| Adjusted | 1.70(1.54-1.87) | Ref | 1.22(0.95-1.58) | 3.28(2.43-4.43) |
| Data are presented as OR(95% CI).  OR = odds ratio, CI = confidence interval, Ref = reference.  Unadjusted: included fibrinogen without adjustment for covariates;  Adjusted: adjusted for age, heart failure and liver disease, renal disease, chronic pulmonary disease, HR, RR, SpO2, SBP, DBP, MBP, PT, APTT, INR, BUN, creatinine, sodium, potassium, hemoglobin, hematocrit, PLT, RBC, RDW, WBC, lactate, total bilirubin, ALT, AST. | | | | |

| 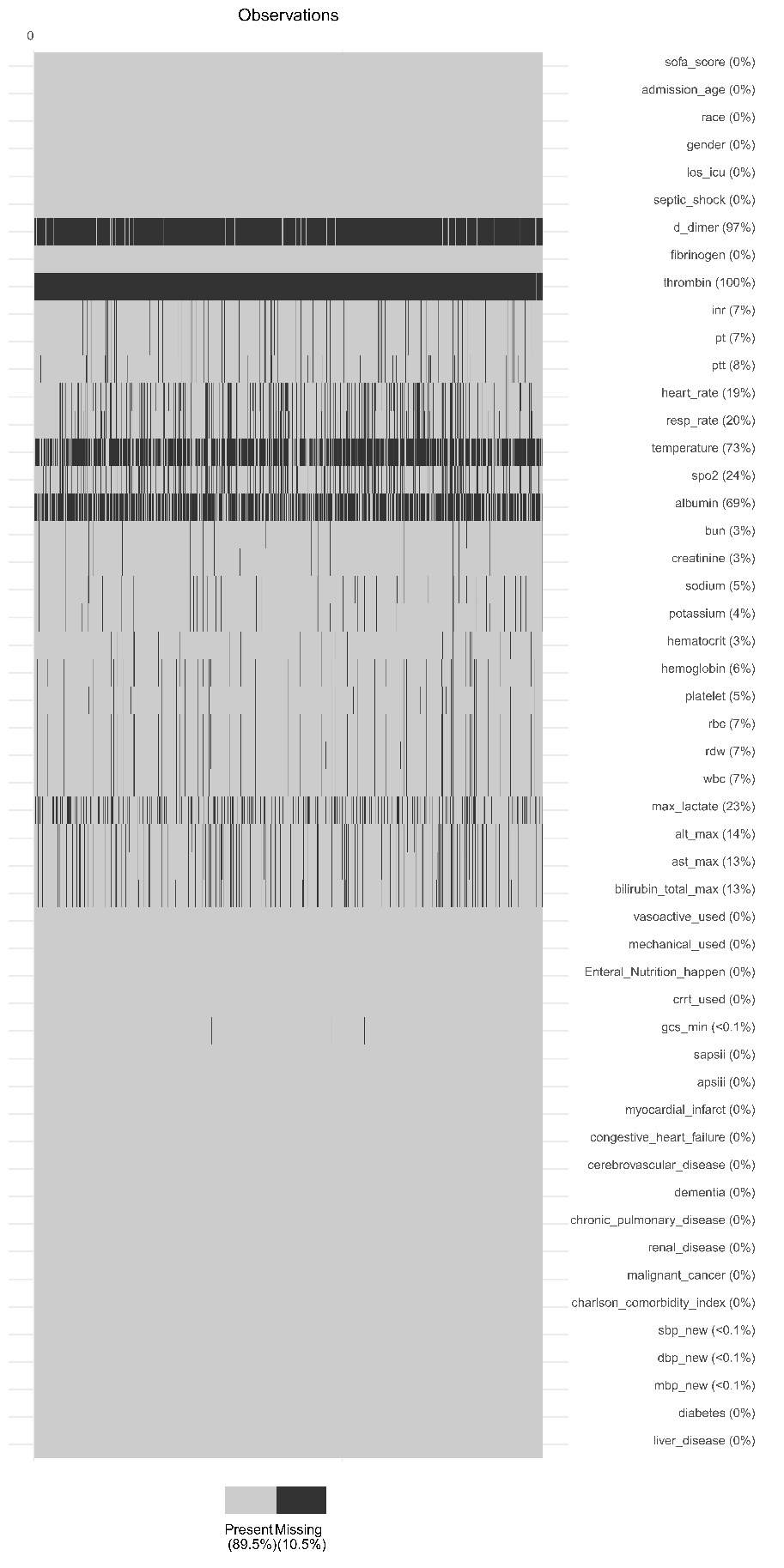 |
| --- |
| Supplementary Fig. 1. The miss rate of feature extraction in MIMIC-IV databases.  Abbreviations: ICU LOS, icu length of stay; COPD, chronic obstructive pulmonary disease; INR, international normalized ratio; PT, prothrombin time; APTT, activated partial thromboplastin time; HR, heart rate; bpm, beats per minute; RR, respiratory rate; SBP, systolic blood pressure; DBP, diastolic blood pressure; MBP, mean blood pressure; SpO_2,_ blood oxygen saturation; BUN, blood urea nitrogen; RBC, red blood cell; RDW, red cell distribution width; WBC, white blood cell; PLT, platelet; ALT, alanine aminotransferase; AST, aspartate aminotransferase; CRRT, Continuous Renal Replacement Therapy; GCS, Glasgow Coma Score; SAPS II, Simplified Acute Physiology Score; APS III, Acute Physiology Score III; CCI, Charlson Comorbidity Index; SOFA, Sequential Organ Failure Assessment.  One black block represented one missing value for each feature. |

| **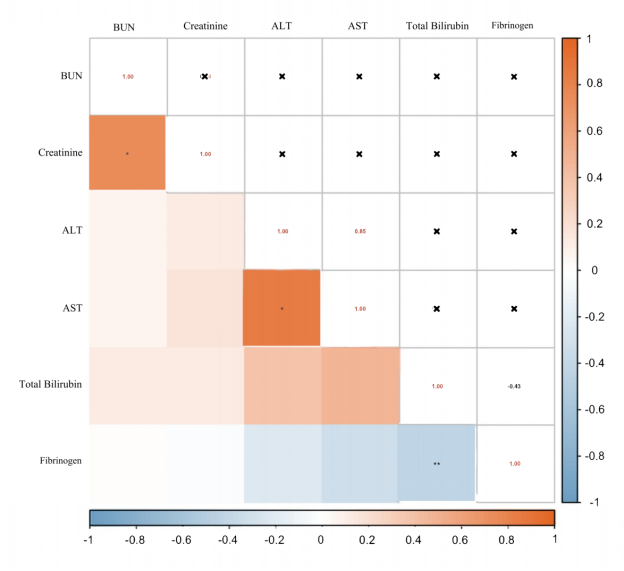** |
| --- |
| Supplementary Fig. 2. Spearman correlation among fibrinogen and BUN, creatine, ALT, AST, total bilirubin.  Abbreviations: BUN, blood urea nitrogen; ALT, alanine aminotransferase; AST, aspartate aminotransferase |
